# Supplementary figures and images for: Transcriptional Landscape of Ectomycorrhizal Fungi and Their Host Provides Insight into N Uptake from Forest Soil
Source: mSystems. 2022 Jan 4;7(1):e00957-21. doi: 10.1128/mSystems.00957-21 (PMC8725588; doi:10.1128/mSystems.00957-21)

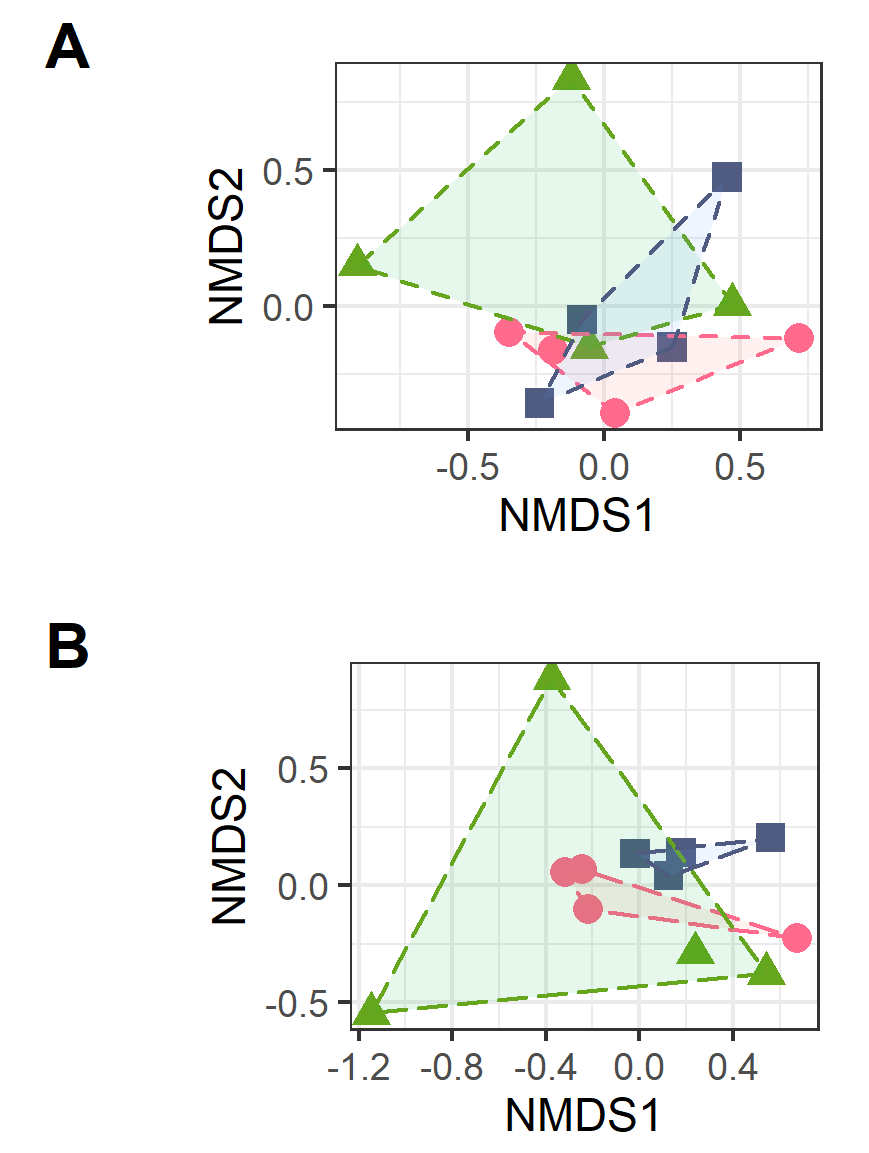

Supplement: FIG S1 [file msystems.00957-21-sf001.tif]

A

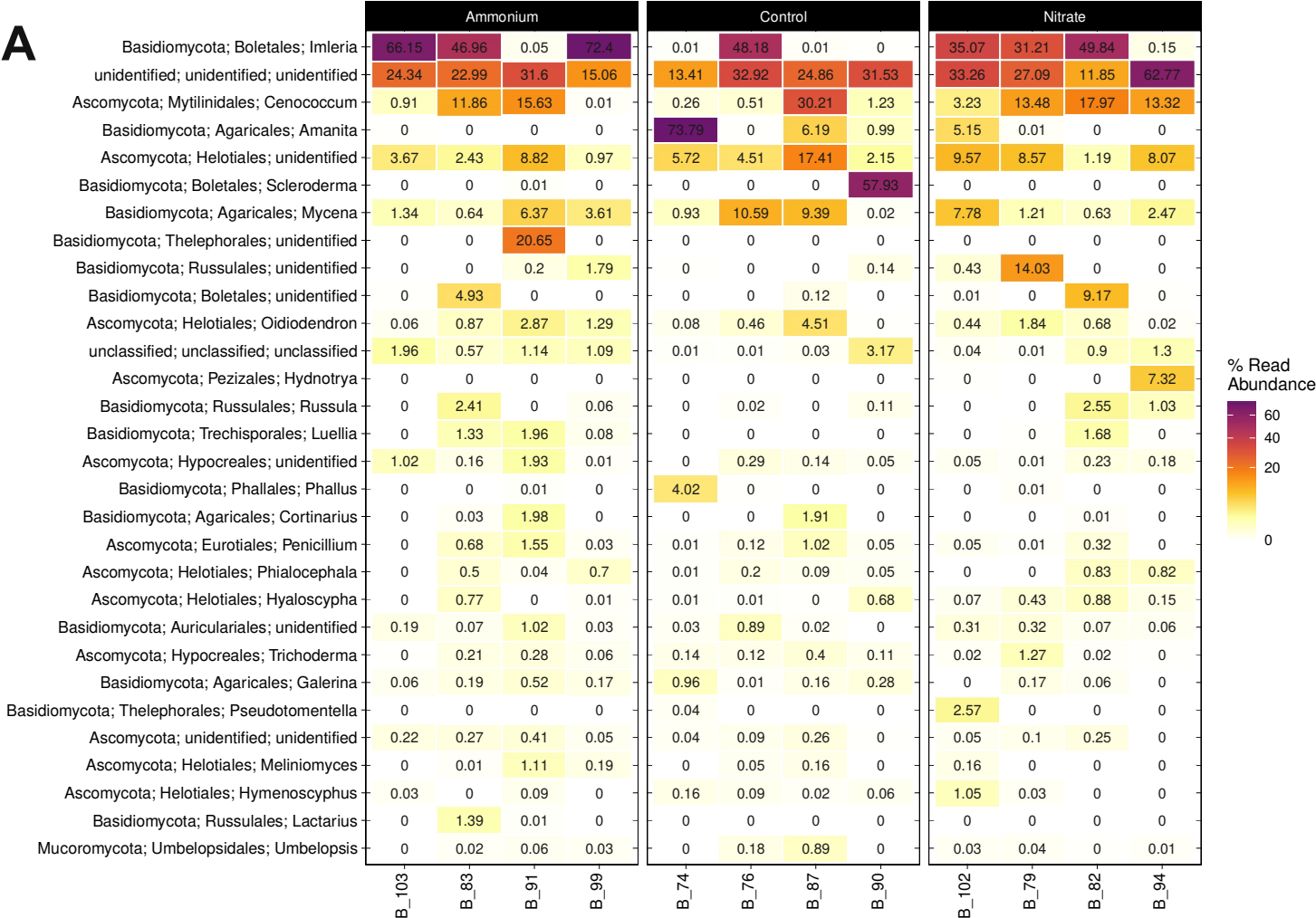

B

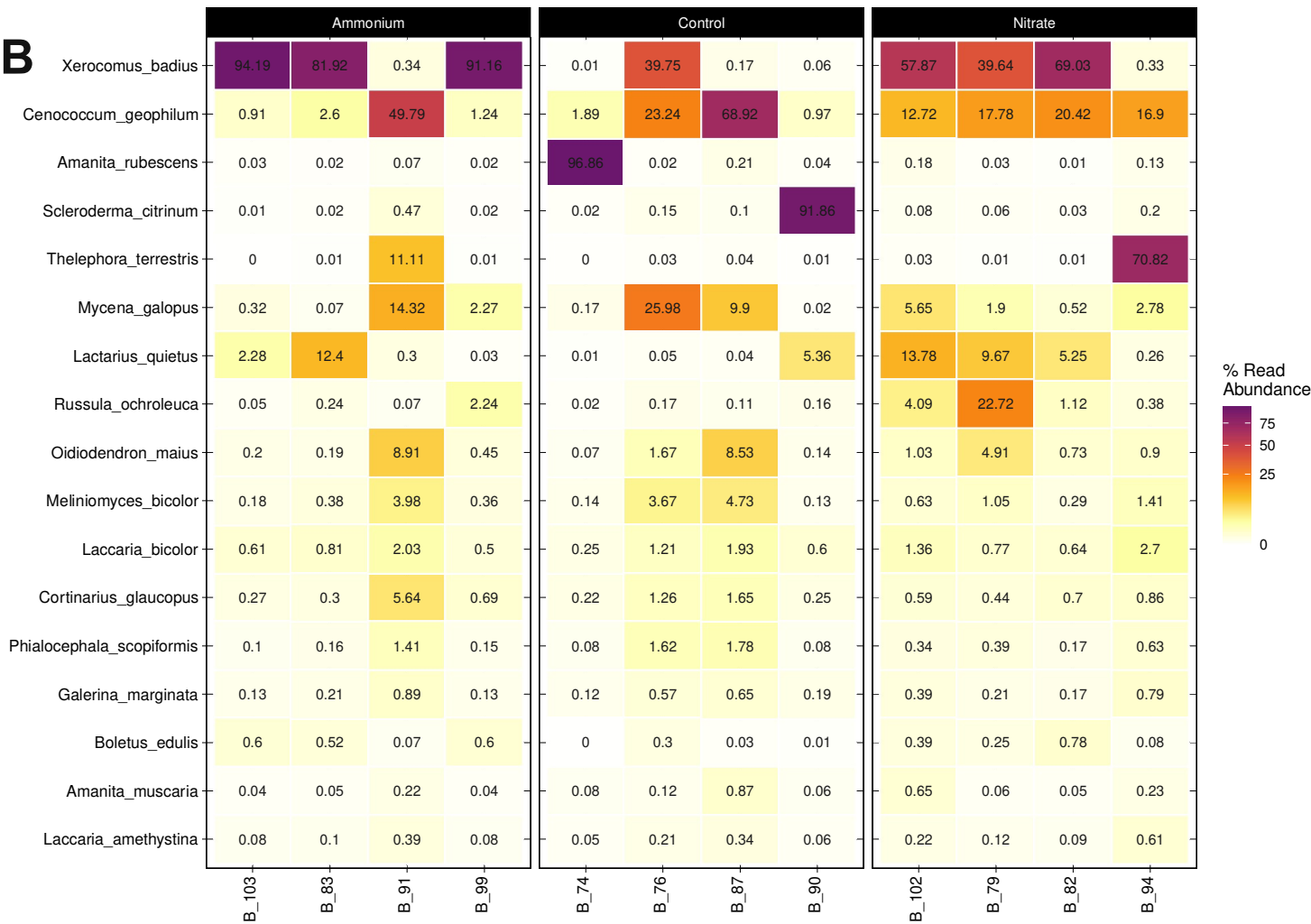

Supplement: FIG S2 [file msystems.00957-21-sf002.pdf]
