# Supplementary material for: Transcriptional Landscape of Ectomycorrhizal Fungi and Their Host Provides Insight into N Uptake from Forest Soil
Source: mSystems. 2022 Jan 4;7(1):e00957-21. doi: 10.1128/mSystems.00957-21 (PMC8725588; doi:10.1128/mSystems.00957-21)
Supplement: TABLE S1 [file msystems.00957-21-st001.docx]

**TABLE S1**

| **Sample** | **Treatment** | **Taxa S** | **Dominance D** | **Simpson 1-D** | **Shannon H** | **Evenness e^H/S** | **Equitability J** | **Fisher alpha** | **Berger-Parker** | **Chao-1** |
| --- | --- | --- | --- | --- | --- | --- | --- | --- | --- | --- |
| B_74 | demineralized water | 65 | 0.5556 | 0.4444 | 1.198 | 0.05095 | 0.2869 | 8.35 | 0.7379 | 75.11 |
| B_76 | demineralized water | 105 | 0.2972 | 0.7028 | 1.775 | 0.05621 | 0.3815 | 14.52 | 0.4817 | 127.6 |
| B_87 | demineralized water | 123 | 0.1523 | 0.8477 | 2.462 | 0.09534 | 0.5116 | 17.45 | 0.3021 | 139.2 |
| B_90 | demineralized water | 72 | 0.3935 | 0.6065 | 1.468 | 0.06026 | 0.3431 | 9.391 | 0.5712 | 96.43 |
| B_83 | 19.85 mM ^15^NH_4_Cl | 111 | 0.2567 | 0.7433 | 2.135 | 0.07621 | 0.4534 | 15.49 | 0.4694 | 165.1 |
| B_91 | 19.85 mM ^15^NH_4_Cl | 135 | 0.09471 | 0.9053 | 2.929 | 0.1385 | 0.597 | 19.46 | 0.2065 | 146.3 |
| B_99 | 19.85 mM ^15^NH_4_Cl | 118 | 0.5295 | 0.4705 | 1.454 | 0.03628 | 0.3048 | 16.63 | 0.7237 | 180 |
| B_103 | 19.85 mM ^15^NH_4_Cl | 55 | 0.4634 | 0.5366 | 1.375 | 0.0719 | 0.3431 | 6.896 | 0.6612 | 66.67 |
| B_79 | 19.98 mM ^15^KNO_3_ | 83 | 0.1786 | 0.8214 | 2.128 | 0.1012 | 0.4816 | 11.06 | 0.312 | 99.87 |
| B_82 | 19.98 mM ^15^KNO_3_ | 98 | 0.2927 | 0.7073 | 1.911 | 0.06899 | 0.4168 | 13.4 | 0.498 | 149.7 |
| B_94 | 19.98 mM ^15^KNO_3_ | 105 | 0.3535 | 0.6465 | 1.793 | 0.05721 | 0.3853 | 14.52 | 0.5708 | 125.3 |
| B_102 | 19.98 mM ^15^KNO_3_ | 126 | 0.1616 | 0.8384 | 2.495 | 0.0962 | 0.5159 | 17.95 | 0.3505 | 140.5 |
