# Supplementary material for: Transcriptional Landscape of Ectomycorrhizal Fungi and Their Host Provides Insight into N Uptake from Forest Soil
Source: mSystems. 2022 Jan 4;7(1):e00957-21. doi: 10.1128/mSystems.00957-21 (PMC8725588; doi:10.1128/mSystems.00957-21)
Supplement: TABLE S2 [file msystems.00957-21-st002.docx]

**TABLE S2**

| **Nr.** | **Pathway map (number of matched objects)** |
| --- | --- |
| 1 | lbc01100 Metabolic pathways - Laccaria bicolor (538) |
| 2 | lbc01110 Biosynthesis of secondary metabolites - Laccaria bicolor (245) |
| 3 | **lbc01230 Biosynthesis of amino acids - Laccaria bicolor (90)** |
| 4 | **lbc01200 Carbon metabolism - Laccaria bicolor (83)** |
| 5 | lbc00230 Purine metabolism - Laccaria bicolor (44) |
| 6 | lbc00520 Amino sugar and nucleotide sugar metabolism - Laccaria bicolor (43) |
| 7 | lbc00500 Starch and sucrose metabolism - Laccaria bicolor (37) |
| 8 | lbc00010 Glycolysis / Gluconeogenesis - Laccaria bicolor (35) |
| 9 | lbc00270 Cysteine and methionine metabolism - Laccaria bicolor (34) |
| 10 | lbc00620 Pyruvate metabolism - Laccaria bicolor (32) |
| 11 | lbc00564 Glycerophospholipid metabolism - Laccaria bicolor (31) |
| 12 | lbc00240 Pyrimidine metabolism - Laccaria bicolor (29) |
| 13 | **lbc00250 Alanine, aspartate and glutamate metabolism - Laccaria bicolor (29)** |
| 14 | lbc00970 Aminoacyl-tRNA biosynthesis - Laccaria bicolor (29) |
| 15 | lbc00561 Glycerolipid metabolism - Laccaria bicolor (28) |
| 16 | lbc00280 Valine, leucine and isoleucine degradation - Laccaria bicolor (27) |
| 17 | lbc00480 Glutathione metabolism - Laccaria bicolor (27) |
| 18 | lbc01210 2-Oxocarboxylic acid metabolism - Laccaria bicolor (27) |
| 19 | lbc00260 Glycine, serine and threonine metabolism - Laccaria bicolor (25) |
| 20 | lbc00020 Citrate cycle (TCA cycle) - Laccaria bicolor (24) |
| 21 | **lbc00330 Arginine and proline metabolism - Laccaria bicolor (23)** |
| 22 | lbc04146 Peroxisome - Laccaria bicolor (23) |
| 23 | lbc00030 Pentose phosphate pathway - Laccaria bicolor (22) |
| 24 | lbc00071 Fatty acid degradation - Laccaria bicolor (22) |
| 25 | lbc00380 Tryptophan metabolism - Laccaria bicolor (21) |
| 26 | lbc00562 Inositol phosphate metabolism - Laccaria bicolor (21) |
| 27 | lbc00630 Glyoxylate and dicarboxylate metabolism - Laccaria bicolor (21) |
| 28 | lbc00051 Fructose and mannose metabolism - Laccaria bicolor (18) |
| 29 | lbc00310 Lysine degradation - Laccaria bicolor (18) |
| 30 | lbc00770 Pantothenate and CoA biosynthesis - Laccaria bicolor (17) |
| 31 | **lbc00220 Arginine biosynthesis - Laccaria bicolor (16)** |
| 32 | lbc03050 Proteasome - Laccaria bicolor (16) |
| 33 | lbc01212 Fatty acid metabolism - Laccaria bicolor (15) |
| 34 | lbc03410 Base excision repair - Laccaria bicolor (15) |
| 35 | lbc04070 Phosphatidylinositol signaling system - Laccaria bicolor (15) |
| 36 | lbc00040 Pentose and glucuronate interconversions - Laccaria bicolor (14) |
| 37 | lbc00340 Histidine metabolism - Laccaria bicolor (14) |
| 38 | lbc00350 Tyrosine metabolism - Laccaria bicolor (14) |
| 39 | lbc00410 beta-Alanine metabolism - Laccaria bicolor (14) |
| 40 | lbc00680 Methane metabolism - Laccaria bicolor (14) |
| 41 | lbc00053 Ascorbate and aldarate metabolism - Laccaria bicolor (13) |
| 42 | lbc00640 Propanoate metabolism - Laccaria bicolor (13) |
| 43 | lbc03030 DNA replication - Laccaria bicolor (13) |
| 44 | lbc00400 Phenylalanine, tyrosine and tryptophan biosynthesis - Laccaria bicolor (12) |
| 45 | lbc00510 N-Glycan biosynthesis - Laccaria bicolor (12) |
| 46 | lbc00600 Sphingolipid metabolism - Laccaria bicolor (12) |
| 47 | lbc00650 Butanoate metabolism - Laccaria bicolor (12) |
| 48 | lbc00860 Porphyrin and chlorophyll metabolism - Laccaria bicolor (12) |
| 49 | lbc00920 Sulfur metabolism - Laccaria bicolor (12) |
| 50 | lbc04138 Autophagy - yeast - Laccaria bicolor (12) |
| 51 | lbc00300 Lysine biosynthesis - Laccaria bicolor (11) |
| 52 | lbc00760 Nicotinate and nicotinamide metabolism - Laccaria bicolor (11) |
| 53 | lbc00052 Galactose metabolism - Laccaria bicolor (10) |
| 54 | lbc00670 One carbon pool by folate - Laccaria bicolor (10) |
| 55 | lbc04011 MAPK signaling pathway - yeast - Laccaria bicolor (10) |
| 56 | lbc00740 Riboflavin metabolism - Laccaria bicolor (9) |
| 57 | lbc00130 Ubiquinone and other terpenoid-quinone biosynthesis - Laccaria bicolor (8) |
| 58 | lbc00360 Phenylalanine metabolism - Laccaria bicolor (8) |
| 59 | lbc00450 Selenocompound metabolism - Laccaria bicolor (8) |
| 60 | lbc00900 Terpenoid backbone biosynthesis - Laccaria bicolor (8) |
| 61 | **lbc00910 Nitrogen metabolism - Laccaria bicolor (8)** |
| 62 | lbc03008 Ribosome biogenesis in eukaryotes - Laccaria bicolor (8) |
| 63 | lbc03015 mRNA surveillance pathway - Laccaria bicolor (8) |
| 64 | lbc04113 Meiosis - yeast - Laccaria bicolor (8) |
| 65 | lbc00061 Fatty acid biosynthesis - Laccaria bicolor (7) |
| 66 | lbc00290 Valine, leucine and isoleucine biosynthesis - Laccaria bicolor (7) |
| 67 | lbc00430 Taurine and hypotaurine metabolism - Laccaria bicolor (7) |
| 68 | lbc00460 Cyanoamino acid metabolism - Laccaria bicolor (7) |
| 69 | lbc00513 Various types of N-glycan biosynthesis - Laccaria bicolor (7) |
| 70 | lbc00730 Thiamine metabolism - Laccaria bicolor (7) |
| 71 | lbc03013 RNA transport - Laccaria bicolor (7) |
| 72 | lbc03018 RNA degradation - Laccaria bicolor (7) |
| 73 | lbc04139 Mitophagy - yeast - Laccaria bicolor (7) |
| 74 | lbc00100 Steroid biosynthesis - Laccaria bicolor (6) |
| 75 | lbc00511 Other glycan degradation - Laccaria bicolor (6) |
| 76 | lbc00780 Biotin metabolism - Laccaria bicolor (6) |
| 77 | lbc00790 Folate biosynthesis - Laccaria bicolor (6) |
| 78 | lbc03420 Nucleotide excision repair - Laccaria bicolor (6) |
| 79 | lbc01040 Biosynthesis of unsaturated fatty acids - Laccaria bicolor (5) |
| 80 | lbc03020 RNA polymerase - Laccaria bicolor (5) |
| 81 | lbc04111 Cell cycle - yeast - Laccaria bicolor (5) |
| 82 | lbc04141 Protein processing in endoplasmic reticulum - Laccaria bicolor (5) |
| 83 | lbc04144 Endocytosis - Laccaria bicolor (5) |
| 84 | lbc00190 Oxidative phosphorylation - Laccaria bicolor (4) |
| 85 | lbc00261 Monobactam biosynthesis - Laccaria bicolor (4) |
| 86 | lbc00592 alpha-Linolenic acid metabolism - Laccaria bicolor (4) |
| 87 | lbc00750 Vitamin B6 metabolism - Laccaria bicolor (4) |
| 88 | lbc03450 Non-homologous end-joining - Laccaria bicolor (4) |
| 89 | lbc00062 Fatty acid elongation - Laccaria bicolor (3) |
| 90 | lbc00072 Synthesis and degradation of ketone bodies - Laccaria bicolor (3) |
| 91 | lbc00514 Other types of O-glycan biosynthesis - Laccaria bicolor (3) |
| 92 | lbc00515 Mannose type O-glycan biosynthesis - Laccaria bicolor (3) |
| 93 | lbc00565 Ether lipid metabolism - Laccaria bicolor (3) |
| 94 | lbc00590 Arachidonic acid metabolism - Laccaria bicolor (3) |
| 95 | lbc03040 Spliceosome - Laccaria bicolor (3) |
| 96 | lbc03430 Mismatch repair - Laccaria bicolor (3) |
| 97 | lbc00332 Carbapenem biosynthesis - Laccaria bicolor (2) |
| 98 | lbc00603 Glycosphingolipid biosynthesis - globo and isoglobo series - Laccaria bicolor (2) |
| 99 | lbc03060 Protein export - Laccaria bicolor (2) |
| 100 | lbc04136 Autophagy - other - Laccaria bicolor (2) |
| 101 | lbc00531 Glycosaminoglycan degradation - Laccaria bicolor (1) |
| 102 | lbc00563 Glycosylphosphatidylinositol (GPI)-anchor biosynthesis - Laccaria bicolor (1) |
| 103 | lbc00604 Glycosphingolipid biosynthesis - ganglio series - Laccaria bicolor (1) |
| 104 | lbc00660 C5-Branched dibasic acid metabolism - Laccaria bicolor (1) |
| 105 | lbc03440 Homologous recombination - Laccaria bicolor (1) |
| 106 | lbc04120 Ubiquitin mediated proteolysis - Laccaria bicolor (1) |
| 107 | lbc04122 Sulfur relay system - Laccaria bicolor (1) |
| 108 | lbc04145 Phagosome - Laccaria bicolor (1) |
