# Supplementary material for: Transcriptional Landscape of Ectomycorrhizal Fungi and Their Host Provides Insight into N Uptake from Forest Soil
Source: mSystems. 2022 Jan 4;7(1):e00957-21. doi: 10.1128/mSystems.00957-21 (PMC8725588; doi:10.1128/mSystems.00957-21)
Supplement: TABLE S3 [file msystems.00957-21-st003.docx]

**TABLE S3**

| **GO Accession** | **Ontology** | **Description** | **P value** |
| --- | --- | --- | --- |
| GO:0015103 | MF | inorganic anion transmembrane transporter activity | 0.000475611 |
| GO:0015112 | MF | nitrate transmembrane transporter activity | 0.001682898 |
| GO:0015318 | MF | inorganic molecular entity transmembrane transporter activity | 0.02315464 |
| GO:0008509 | MF | anion transmembrane transporter activity | 0.02315464 |
| GO:0015075 | MF | ion transmembrane transporter activity | 0.02315464 |
| GO:0008308 | MF | voltage-gated anion channel activity | 0.025644629 |
| GO:0005216 | MF | ion channel activity | 0.031224718 |
| GO:0005253 | MF | anion channel activity | 0.031224718 |
| GO:0005244 | MF | voltage-gated ion channel activity | 0.032827156 |
| GO:0016710 | MF | trans-cinnamate 4-monooxygenase activity | 0.032827156 |
| GO:0019150 | MF | D-ribulokinase activity | 0.032827156 |
| GO:0042300 | MF | beta-amyrin synthase activity | 0.032827156 |
| GO:0009671 | MF | nitrate:proton symporter activity | 0.032827156 |
| GO:0008909 | MF | isochorismate synthase activity | 0.032827156 |
| GO:0016712 | MF | oxidoreductase activity, acting on paired donors, with incorporation or reduction of molecular oxygen, reduced flavin or flavoprotein as one donor, and incorporation of one atom of oxygen | 0.032827156 |
| GO:0015267 | MF | channel activity | 0.032827156 |
| GO:0022832 | MF | voltage-gated channel activity | 0.032827156 |
| GO:0022803 | MF | passive transmembrane transporter activity | 0.032827156 |
| GO:0062047 | MF | pipecolic acid N-hydroxylase | 0.032827156 |
| GO:0098809 | MF | nitrite reductase activity | 0.032827156 |
| GO:0017096 | MF | acetylserotonin O-methyltransferase activity | 0.032827156 |
| GO:0016662 | MF | oxidoreductase activity, acting on other nitrogenous compounds as donors, cytochrome as acceptor | 0.032827156 |
| GO:0050421 | MF | nitrite reductase (NO-forming) activity | 0.032827156 |
| GO:0019825 | MF | oxygen binding | 0.041848523 |
| GO:0016211 | MF | ammonia ligase activity | 0.041848523 |
| GO:0080019 | MF | fatty-acyl-CoA reductase (alcohol-forming) activity | 0.041848523 |
| GO:0015296 | MF | anion:cation symporter activity | 0.041848523 |
| GO:0004356 | MF | glutamate-ammonia ligase activity | 0.041848523 |
| GO:0015098 | MF | molybdate ion transmembrane transporter activity | 0.041848523 |
| GO:0031559 | MF | oxidosqualene cyclase activity | 0.041848523 |
| GO:0015513 | MF | high-affinity secondary active nitrite transmembrane transporter activity | 0.041848523 |
| GO:0004016 | MF | adenylate cyclase activity | 0.041848523 |
| GO:0004820 | MF | glycine-tRNA ligase activity | 0.041848523 |
| GO:0004345 | MF | glucose-6-phosphate dehydrogenase activity | 0.041848523 |
| GO:0050486 | MF | intramolecular transferase activity, transferring hydroxy groups | 0.041848523 |
| GO:0022857 | MF | transmembrane transporter activity | 0.041848523 |
| GO:0022836 | MF | gated channel activity | 0.045553402 |
| GO:0016866 | MF | intramolecular transferase activity | 0.046986661 |
| GO:0015698 | BP | inorganic anion transport | 0.000685462 |
| GO:0010167 | BP | response to nitrate | 0.003443921 |
| GO:0015706 | BP | nitrate transport | 0.003443921 |
| GO:0006821 | BP | chloride transport | 0.03664734 |
