# Supplementary material for: Transcriptional Landscape of Ectomycorrhizal Fungi and Their Host Provides Insight into N Uptake from Forest Soil
Source: mSystems. 2022 Jan 4;7(1):e00957-21. doi: 10.1128/mSystems.00957-21 (PMC8725588; doi:10.1128/mSystems.00957-21)
Supplement: TABLE S4 [file msystems.00957-21-st004.docx]

**TABLE S4**

| Date | Air temperature (°C) | Air humidity (%) |
| --- | --- | --- |
| 17 July 2018 to 19 July 2018 | 22.8 ± 4.47 | 52.57 ± 8.32 |
| 31 July 2018 to 2 August 2018 | 27.16 ± 3.58 | 54.70 ± 11.77 |
| March | 4.72 ± 5.00 | 78.34 ± 16.04 |
| April | 14.16 ± 5.86 | 68.10 ± 18.53 |
| May | 17.74 ± 6.05 | 64.45 ± 19.12 |
| June | 18.87 ± 5.16 | 70.70 ± 17.76 |
| July | 22.40 ± 6.46 | 57.99 ± 20.59 |
| August | 26.34 ± 4.70 | 57.83 ± 14.81 |
