# Supplementary material for: Transcriptional Landscape of Ectomycorrhizal Fungi and Their Host Provides Insight into N Uptake from Forest Soil
Source: mSystems. 2022 Jan 4;7(1):e00957-21. doi: 10.1128/mSystems.00957-21 (PMC8725588; doi:10.1128/mSystems.00957-21)
Supplement: TABLE S5 [file msystems.00957-21-st005.docx]

**TABLE S5**

| **Sample** | **Treatment** | **Raw reads** | **Filtered reads** | **Processed reads (%)** | **Number of fungal reads** | **Number of fungal OTUs before rarefaction** | **Number of fungal OTUs after rarefaction** |
| --- | --- | --- | --- | --- | --- | --- | --- |
| B_74 | demineralized water | 81455 | 76478 | 93.89 | 47733 | 85 | 65 |
| B_76 | demineralized water | 33591 | 31327 | 93.26 | **20051** | 105 | 105 |
| B_87 | demineralized water | 65622 | 61057 | 93.04 | 32810 | 132 | 123 |
| B_90 | demineralized water | 45123 | 41405 | 91.76 | 26636 | 75 | 72 |
| B_83 | 19.85 mM ^15^NH_4_Cl | 57103 | 53497 | 93.69 | 41015 | 127 | 111 |
| B_91 | 19.85 mM ^15^NH_4_Cl | 86563 | 80679 | 93.20 | 50894 | 159 | 135 |
| B_99 | 19.85 mM ^15^NH_4_Cl | 60363 | 56470 | 93.55 | 32104 | 126 | 118 |
| B_103 | 19.85 mM ^15^NH_4_Cl | 45808 | 43041 | 93.96 | 33304 | 72 | 55 |
| B_79 | 19.98 mM ^15^KNO_3_ | 72175 | 67093 | 92.96 | 40852 | 104 | 83 |
| B_82 | 19.98 mM ^15^KNO_3_ | 48344 | 45565 | 94.25 | 35446 | 111 | 98 |
| B_94 | 19.98 mM ^15^KNO_3_ | 49988 | 46847 | 93.72 | 31601 | 120 | 105 |
| B_102 | 19.98 mM ^15^KNO_3_ | 43122 | 40659 | 94.29 | 31177 | 139 | 126 |
