# Supplementary material for: Transcriptional Landscape of Ectomycorrhizal Fungi and Their Host Provides Insight into N Uptake from Forest Soil
Source: mSystems. 2022 Jan 4;7(1):e00957-21. doi: 10.1128/mSystems.00957-21 (PMC8725588; doi:10.1128/mSystems.00957-21)
Supplement: TABLE S6 [file msystems.00957-21-st006.docx]

**TABLE S6**

| **Sample** | **Treatment** | **RNA Integrity Number** | **Raw reads** | **Processed reads** | **% processed reads** | **Mapped reads** | **Mapped to fungi** | **Mapped to Fagus** | **% mapped** | **% mapped fungi** | **% mapped Fagus** |
| --- | --- | --- | --- | --- | --- | --- | --- | --- | --- | --- | --- |
|  |  |  |  |  |  |  |  |  |  |  |  |
| B_74 | demineralized water | 6.7 | 117,404,490 | 116,514,831 | 99.24 | 75,838,597 | 38,345,820 | 37,492,777 | 65.09 | 32.91 | 32.18 |
| B_76 | demineralized water | 7.0 | 110,533,021 | 109,713,595 | 99.26 | 64,762,941 | 5,678,950 | 59,083,991 | 59.03 | 5.18 | 53.85 |
| B_87 | demineralized water | 7.9 | 123,830,699 | 123,001,454 | 99.33 | 78,074,569 | 3,662,184 | 74,412,385 | 63.47 | 2.98 | 60.5 |
| B_90 | demineralized water | 7.6 | 113,626,927 | 112,848,395 | 99.31 | 73,581,581 | 33,664,910 | 39,916,671 | 65.2 | 29.83 | 35.37 |
| B_83 | 19.85 mM ^15^NH_4_Cl | 7.6 | 113,949,314 | 113,135,195 | 99.29 | 68,432,056 | 29,792,675 | 38,639,381 | 60.49 | 26.33 | 34.15 |
| B_91 | 19.85 mM ^15^NH_4_Cl | 7.4 | 101,039,883 | 100,271,972 | 99.24 | 56,633,842 | 3,005,149 | 53,628,693 | 56.48 | 3 | 53.48 |
| B_99 | 19.85 mM ^15^NH_4_Cl | 7.1 | 111,430,088 | 110,558,237 | 99.22 | 70,563,179 | 18,636,615 | 51,926,564 | 63.82 | 16.86 | 46.97 |
| B_103 | 19.85 mM ^15^NH_4_Cl | 7.5 | 109,734,463 | 108,965,024 | 99.3 | 73,756,153 | 23,338,402 | 50,417,751 | 67.69 | 21.42 | 46.27 |
| B_79 | 19.98 mM ^15^KNO_3_ | 7.3 | 102,541,237 | 101,839,654 | 99.32 | 62,315,939 | 11,130,991 | 51,184,948 | 61.19 | 10.93 | 50.26 |
| B_82 | 19.98 mM ^15^KNO_3_ | 7.0 | 98,286,285 | 97,604,837 | 99.31 | 57,297,015 | 26,933,739 | 30,363,276 | 58.7 | 27.59 | 31.11 |
| B_94 | 19.98 mM ^15^KNO_3_ | 7.8 | 113,990,445 | 113,207,752 | 99.31 | 66,413,204 | 5,254,953 | 61,158,251 | 58.66 | 4.64 | 54.02 |
| B_102 | 19.98 mM ^15^KNO_3_ | 7.1 | 107,765,136 | 107,032,242 | 99.32 | 57,103,575 | 13,922,719 | 43,180,856 | 53.35 | 13.01 | 40.34 |
